# Inline Protein Concentration by Vibratory Single Pass Tangential Flow Filtration

**DOI:** 10.1002/biot.70124

**Published:** 2025-09-19

**Authors:** Km Prottoy Shariar Piash, Ziqiao Wang, Claire MacElroy, Andrew L. Zydney

**Affiliations:** ^1^ Department of Chemical Engineering The Pennsylvania State University University Park Pennsylvania USA

**Keywords:** continuous processing, dynamic filtration, monoclonal antibody, SPTFF, ultrafiltration

## Abstract

Single Pass Tangential Flow Filtration (SPTFF) is increasingly used for inline concentration and final formulation in intensified/continuous processes for monoclonal antibody products. However, these modules typically operate at low feed flux, requiring significant membrane area and often complex internal staging to achieve the desired concentration factor. In this study, a vibration‐assisted SPTFF system was used for inline concentration of soluble protein. The maximum sustainable flux and concentration factor were evaluated under vibratory and non‐vibratory conditions using flux‐stepping experiments. SPTFF performed under vibration was able to achieve single pass concentration factors of 20× at a feed flux of 17.2 L/m^2^/h, while the non‐vibratory system showed rapid fouling at much lower concentration factors. Furthermore, the vibratory module achieved a 6‐fold higher concentration factor compared to a screened channel cassette. Long‐term filtration experiments demonstrated that the vibratory system could concentrate a 20 g/L protein solution to 100 g/L using a single cassette with stable operation for more than 8 h without protein aggregation. This work highlights the potential opportunity to develop vibratory SPTFF systems for intensified bioprocessing.

## Graphical Abstract and Lay Summary

This work provides the first demonstration of a vibration‐assisted filtration module for the inline concentration of protein. The vibratory system can provide higher concentration factors than can be obtained in traditional modules, either as single‐stage systems or in complex multistage systems employing multiple cassettes in series or parallel arrangements. The vibratory system could enable inline protein concentration with simpler and lower cost designs, which would be particularly attractive for intensified and/or continuous downstream processing of monoclonal antibody products.

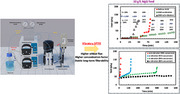

**Abbreviations**
DI: deionized
DLS: dynamic light scattering
hIgG: human serum Immunoglobulin G
mAb: monoclonal antibody
SPTFF: single‐pass tangential flow filtration
TFF: tangential flow filtration
TMP: transmembrane pressure difference

## 1 Introduction

Single‐pass tangential flow filtration (SPTFF) has developed rapidly over the past 10 years as an important unit operation in bioprocessing. In contrast to batch ultrafiltration, in which the product is recirculated through the membrane module multiple times to obtain the desired concentration factor at the end of the process, SPTFF operates at steady‐state with the feed passed through the module only once. The required concentration factor is typically achieved using much lower feed flow rates and by employing a series of membrane cassettes to increase the path length and thus the conversion (equal to the permeate flow rate divided by the feed flow rate).

Most studies of SPTFF have focused on applications in processing of monoclonal antibodies (mAbs) due to the importance of mAb therapeutics; the global market for mAbs is currently more than $200 billion per year [1] with major applications in the treatment of cancer and autoimmune diseases [2, 3]. SPTFF can be used to reduce tank volumes by inline concentration of the eluent from the Protein A chromatography column [4]. SPTFF can pre‐concentrate the feed prior to subsequent chromatography steps, providing more than 2‐fold greater productivity of the Protein A chromatography capture resin [5, 6] and a 4‐fold increase in mAb loading as well as a significant reduction in buffer volume for the anion exchange polisher [7]. SPTFF can also be combined with inline buffer addition to achieve desalting and buffer exchange [8]. Chen et al. [9] have discussed the integration of SPTFF and single‐pass diafiltration in the conversion of a mAb downstream process from batch to continuous operations. More recent work has demonstrated the potential of using SPTFF for intensified or continuous processing in the purification of precipitated proteins [10], mRNA [11], lipid nanoparticles [12], and adeno‐associated virus [13].

One of the challenges in applying SPTFF in commercial mAb processes is the relatively low feed flux (feed flow rate normalized by the membrane area) required to achieve high single‐pass conversions (i.e., high concentration factors) [4]. Membrane manufacturers have developed special SPTFF modules with multiple cassettes in series/parallel arrangements that can be operated with somewhat higher feed flow rates, but these modules tend to be more expensive, and they are available in relatively limited configurations/sizes. For example, Jabra et al. [14] reported that the maximum feed flux that could be used to obtain a 4.5× concentration factor with a 20 g/L Immunoglobulin G feed was < 15 L/m^2^/h using a four‐stage Cadence SPTFF module with seven ultrafiltration cassettes and a total path length of 68 cm. Malladi et al. [15] examined the performance of an SPTFF process employing two Pellicon capsules in series for concentration of a purified mAb product, with a feed flux <1 L/m^2^/h used to concentrate a 5–15 g/L mAb feed to a final concentration of 75 g/L (average concentration factor of 7.5×).

An alternative approach for increasing the flux in membrane processes is to enhance mass transfer (reducing concentration polarization) by external vibration of the membrane module. Jiang et al. [16] achieved as much as 7× higher filtrate flux using a vibratory system for batch ultrafiltration of mixtures of sodium alginate and bovine serum albumin, primarily due to a significant reduction in membrane fouling. Behboudi et al. [11] demonstrated that vibration‐assisted SPTFF could be used for inline concentration of mRNA while Piash et al. [10] showed that vibratory SPTFF can also enhance the flux and conversion during concentration of protein precipitates. However, it is not possible to extrapolate from these studies to the development of SPTFF systems for continuous/intensified processing of mAbs.

The objective of this work was to demonstrate that dynamic vibration‐assisted SPTFF systems can be used for inline protein concentration. Human serum immunoglobulin G (hIgG) was used for all experiments due to its interest in treating immune disorders and its widespread use as a lower‐cost surrogate for mAbs. Flux‐stepping experiments were used to identify the critical filtrate flux, that is, the filtrate flux at which membrane fouling first becomes significant. These data were then used to design an SPTFF process for concentrating a 10–20 g/L hIgG solution by more than 5–8× during 8‐h of continuous operation.

## 2 Materials and Methods

### 2.1 Materials

hIgG was purchased from GoldenWest Diagnostics, LLC (Termecula, CA, USA) and stored at 2°C–8°C. The lyophilized protein was dissolved in 1× Phosphate Buffered Saline (PBS) prepared by dilution of a 10× PBS stock solution (Boston BioProducts, Inc., Milford, MA, USA) with deionized (DI) water. The resulting hIgG solutions were then pre‐filtered through 0.2 µm pore size polyethersulfone membranes in a vacuum filter (Thermo Fisher Scientific, Waltham, MA) to remove any aggregates, with the pre‐filtered hIgG stored at 4°C when not in use.

### 2.2 Filtration Experiments

**FIGURE 1:**
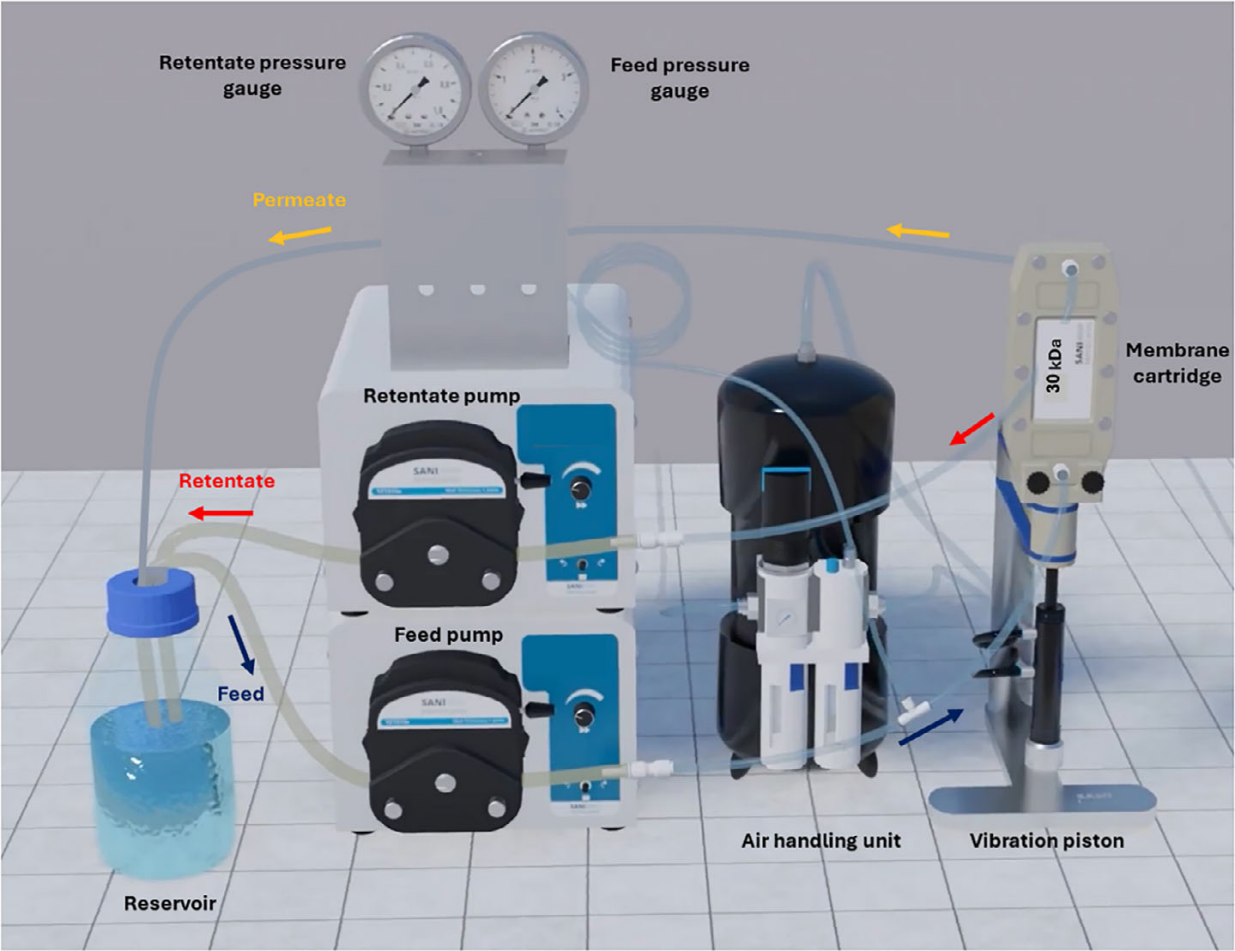
Schematic diagram of Vibro‐Lab35P used in this study. Figure adapted from SANI membranes. Reproduced from [11].

A Vibro‐Lab 35P vibratory membrane filtration system (SANI Membranes, Farum, Denmark) was used for all SPTFF experiments (Figure 1). The open channel module was fitted with a single flat sheet 30 kDa polyethersulfone (PES) membrane (MK MAX, Synder, CA, USA) with an effective surface area of 35 cm^2^. Vibration was generated by a piston actuated by an air handling unit pressurized to approximately 3.4 bar. The membrane module was oriented vertically on the piston, with the feed inlet at the bottom left, the retentate exit at the top left, and the permeate collected through a port positioned opposite to the retentate at the top of the module.

Additional filtration experiments were also performed using the Pellicon XL50 cassette with A‐screen (MilliporeSigma, Burlington, MA, USA) containing 30 kDa Biomax PES membranes. The Pellicon XL50 was mounted vertically in a clamp, with the feed inlet positioned at the lower left and the retentate outlet at the upper left. The permeate port at the lower right was sealed, whereas the permeate outlet at the upper right remained open for timed collection of the permeate flow.

SPTFF experiments were performed at specified filtrate flux, which was controlled using peristaltic pumps (Cole‐Parmer Masterflex L/S) placed on the feed inlet and retentate exit lines; the permeate was at atmospheric pressure. Pressures were evaluated using digital pressure transducers (ESI Technology, UK, GD4200‐USB) at the feed inlet and retentate exit, both connected to a computer for data logging. All experiments were conducted using total recycle, with the retentate and permeate streams recycled to the feed reservoir to minimize the amount of hIgG required for the experiments. Experiments were performed both with and without vibration. After each filtration run, the module was emptied, thoroughly flushed with DI water, cleaned with 0.5 N NaOH at 40°C, and then filled with 0.1 N NaOH to minimize bacterial growth during storage.

The critical flux was determined using the methodology described in prior studies [11, 13]. The module was first flushed with DI water for at least 30 min to remove the storage solution and fully wet the membrane. The hIgG feed (∼150 mL) was then pumped from the solution reservoir at a specified flow rate, with the permeate flow rate increased in a stepwise manner by reducing the retentate flow rate every 25 min. The transmembrane pressure (TMP):

(1) $$TMP=\frac{P_{Feed}+P_{Retentate}}{2}-P_{Permeate}$$

was monitored as a function of time, with the critical flux defined as the average of the filtrate flux values just before and after the TMP became unstable.

Long‑term (continuous) filtration experiments were performed with the feed reservoir containing 800 mL of either a 10 or 20 g/L hIgG solution at a feed flux of 17.2 L/m^2^/h with specified (target) concentration factor.

### 2.3 hIgG Characterization

hIgG concentrations in the feed, permeate, and retentate streams were evaluated by UV absorbance (Tecan Infinite 200 PRO, Tecan Trading AG, Switzerland) at 280 nm based on a calibration curve constructed using hIgG standards from 0–1 g/L. When required, samples were diluted with PBS to maintain the absorbance in the linear regime. No dilution was required for the permeate samples. The viscosity of the hIgG solution was evaluated using a Cannon‐Fenske routine viscometer (Cannon Instrument Company, State College, PA, USA). The possible presence of large hIgG aggregates (>0.1 µm) after SPTFF was examined by Dynamic Light Scattering (Malvern Zetasizer Nano ZS90, Worcestershire, UK).

## 3 Results and Discussion

### 3.1 Flux‐Stepping Experiments

**FIGURE 2:**
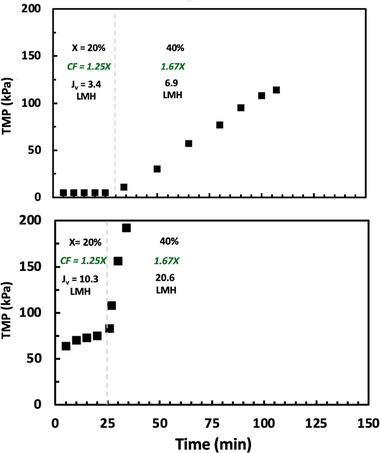
Flux‐stepping experiments performed with 10 g/L solutions of hIgG using 30 kDa polyethersulfone membranes in the Vibro‐Lab 35P open channel cassette without vibration at feed fluxes of 17.2 L/m^2^/h (top panel) and 51.5 L/m^2^/h (bottom panel). The conversion (X), concentration factor (CF), and filtrate flux (J_v_) are shown for each time interval.

Figure 2 shows results for two experiments performed using the flux‐stepping methodology in the Vibro‐Lab 35P open channel cassette, but without any vibration. Data were obtained with 10 g/L solutions of hIgG at feed flow rates of 1 and 3 mL/min, which correspond to feed fluxes of 17.2 and 51.5 L/m^2^/h, respectively. The 30 kDa membranes were fully retentive to the hIgG, with hIgG concentrations in permeate samples below the limit of quantification (corresponding to >99.5% retention).

The TMP in the first step, corresponding to a conversion of 20% (concentration factor of 1.25×), remained stable at both feed flow rates. However, increasing the filtrate flux (by reducing the retentate exit flow rate) to achieve a conversion of *X* = 40% caused a large increase in TMP, particularly for the data at 3 mL/min (lower panel), with the TMP gradient for both feed flow rates becoming greater than 1.3 kPa/min. The TMP thus became unstable at a flux between 3.4 and 6.9 L/m^2^/h at a feed flow rate of 1 mL/min and between J_crit_ = 10.3 and 20.6 L/m^2^/h at 3 mL/min. In both cases, the maximum conversion (*X*_max_) was only between 20% and 40%, providing less than a 2‐fold concentration factor for the 10 g/L hIgG feed.

**FIGURE 3:**
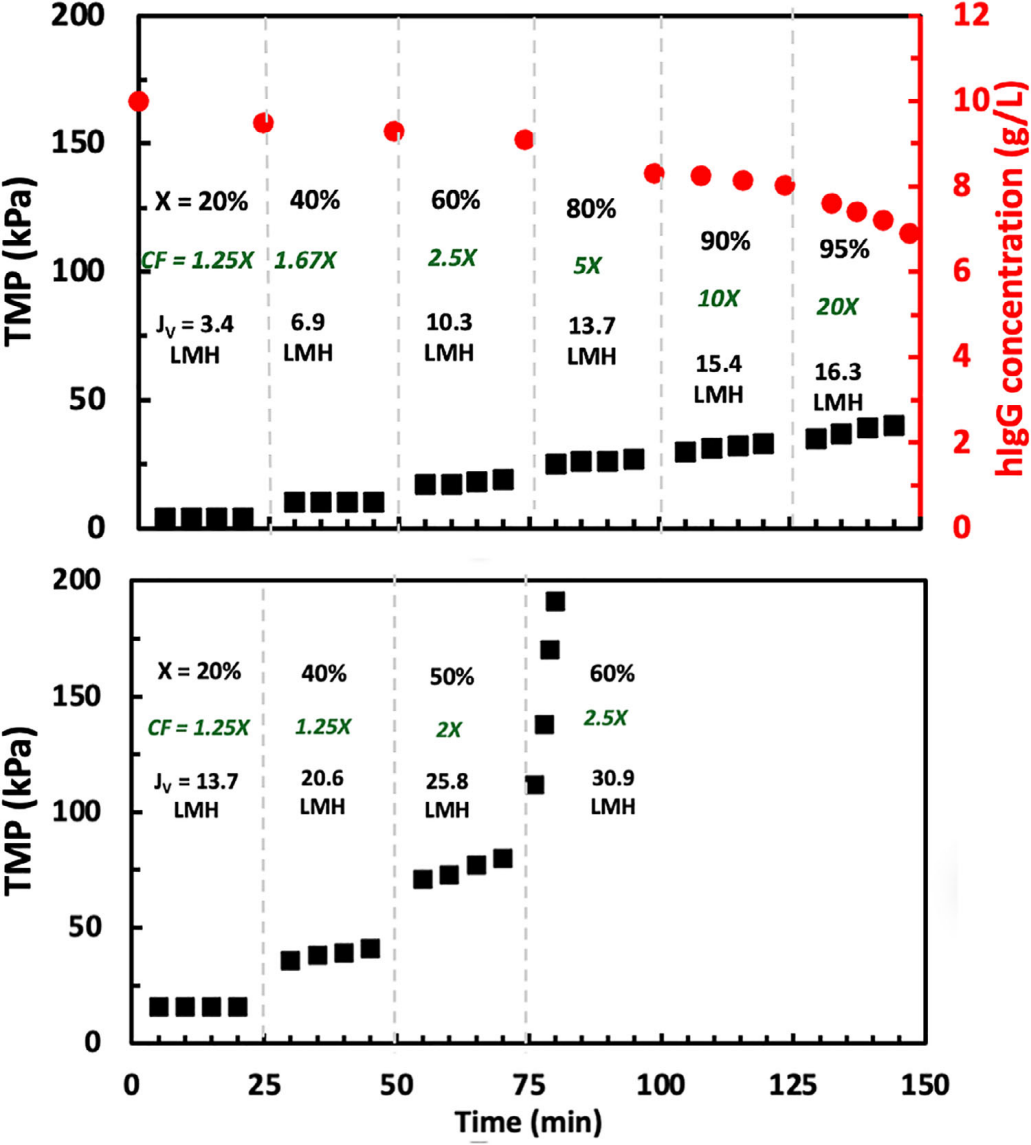
Flux‐stepping experiments performed with 10 g/L solutions of hIgG in the Vibro‐Lab 35P open channel cassette with 30 kDa polyethersulfone UF membrane in the presence of vibration at feed fluxes of 17.2 L/m^2^/h (top panel) and 51.5 L/m^2^/h (bottom panel). The conversion (X), concentration factor (CF), and filtrate flux (J_v_) are shown for each time interval. The red circles in the top panel show the hIgG concentration in the feed reservoir throughout the experiment.

The results from a corresponding series of experiments using the same membrane cassette (after cleaning with 0.5 N NaOH) but in the presence of vibration at a frequency of 26 Hz and an amplitude of approximately 3.5 mm are shown in Figure 3. In this case, the TMP at a feed flux 17.2 L/m^2^/h (top panel) remained stable up to a filtrate (permeate) flux of at least 16.3 L/m^2^/h, corresponding to a maximum conversion of X_max_ > 95% and a maximum concentration factor above 20×. Note that we were unable to identify the critical flux at this feed flow rate since it was not possible to stably operate the retentate pump at flow rates below 0.05 mL/min (0.86 L/m^2^/h). The TMP at a feed flux 51.5 L/m^2^/h remained relatively constant over the first 3 flux steps, but there was a significant increase in TMP when the flux was increased to 30.9 L/m^2^/h. Although the critical flux at this higher feed flux was at least J_crit_ = 25.8 L/m^2^/h, the stable conversion was only 50% (maximum concentration factor of 2×).

The right‐hand axis in the top panel of Figure 3 shows the hIgG concentration in the feed reservoir throughout the flux‐stepping experiment for the 17.2 L/m^2^/h feed flux. The concentration of hIgG in the feed reservoir decreased slightly right at the start of the experiment due to a small dilution effect associated with the buffer volume in the module and tubing. There was a much more significant reduction in the feed concentration at higher conversions due to the relatively large hold‐up volume within the module (approximately 6 mL) and retentate exit line (approximately 1.5 mL) compared to the ~140 mL in the feed reservoir. Thus, the data at 10× concentration factor produced an hIgG product with a concentration of around 82 g/L, while the 20× concentration factor yielded a retentate product that had an hIgG concentration of 140 g/L. A simple mass balance, assuming that the average concentration within the module was equal to the mean between the values in the feed inlet and retentate outlet, was able to account for all of the hIgG in the system (within ± 10%), suggesting that there was negligible deposition of hIgG on the membrane surface (in terms of total hIgG mass in the system).

**FIGURE 4:**
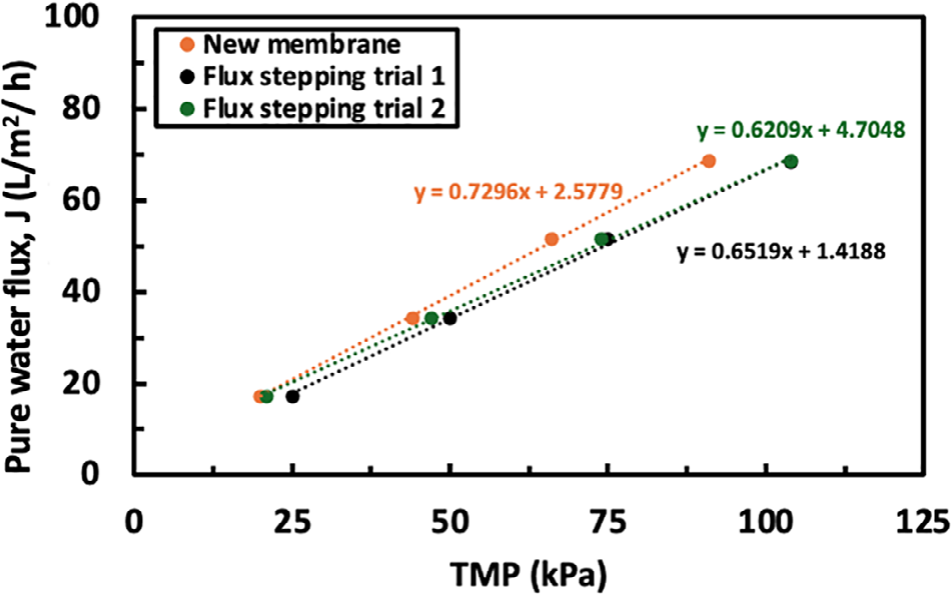
Pure water flux as a function of transmembrane pressure for a new 30 kDa polyethersulfone membrane and for the same membrane after repeat flux‐stepping experiments with cleaning performed using 0.5 M NaOH at 40°C.

Figure 4 shows data for the flux of DI water through the new membrane and through membranes that were used and then cleaned in two consecutive flux‐stepping experiments. The membrane permeability, determined from the slope of the flux versus TMP data, for the new membrane was 0.73 LMH/kPa. The permeabilities of the used/cleaned membranes were slightly lower; however, the permeabilities remained within 10%–15% of the value for the pristine membrane. Furthermore, the permeability of the cleaned membrane after the first flux‐stepping experiment was comparable to the permeability observed after the second experiment, demonstrating that the fouled membranes can be effectively cleaned with 0.5 N NaOH at 40°C.

**FIGURE 5:**
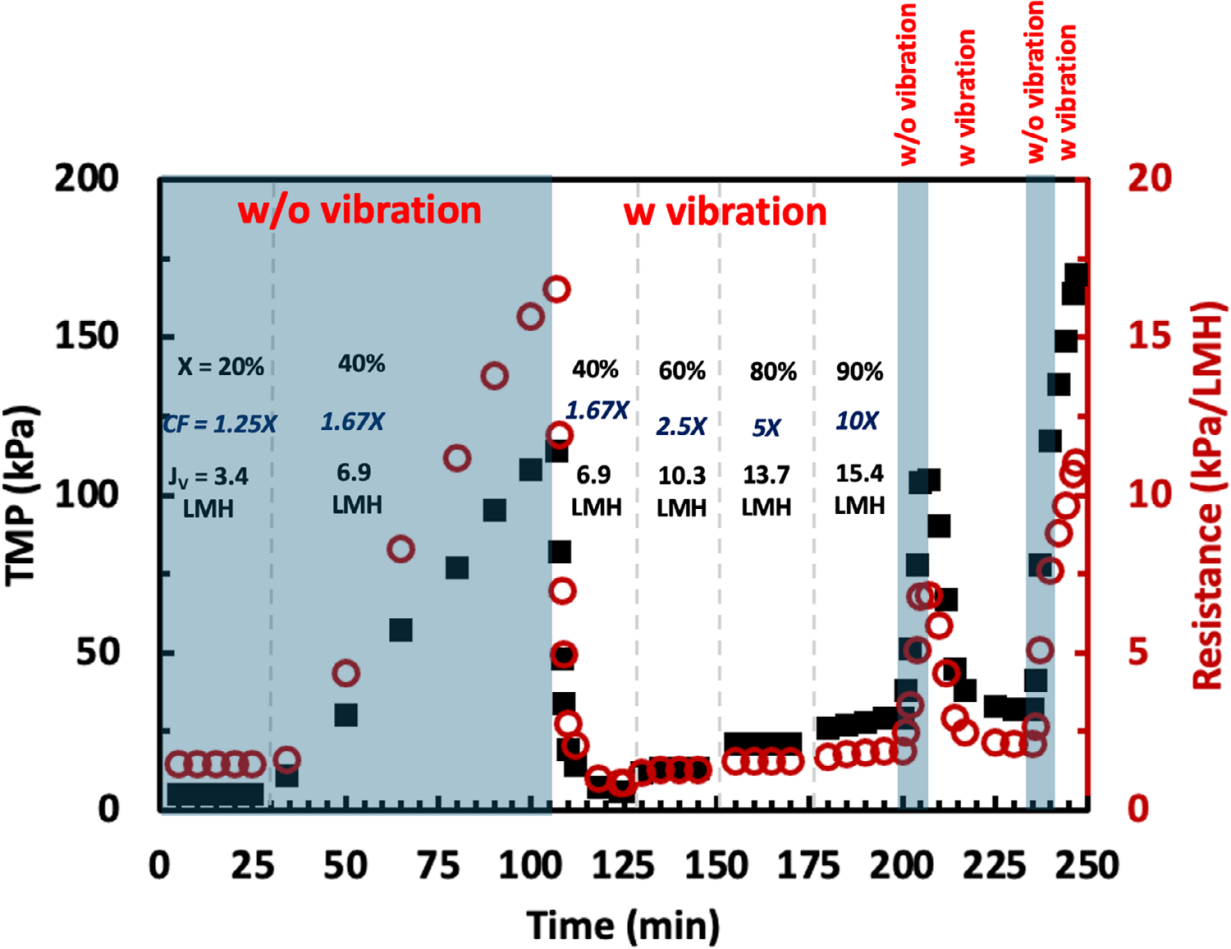
Reversibility of TMP profile upon applying vibration during SPTFF of a 10 g/L solution of hIgG at a feed flux of 17.2 L/m^2^/h. Greyed regions are without vibration, while white regions are with vibration.

The rapid increase in TMP when the flux exceeds J_crit_ is due to a combination of concentration polarization and membrane fouling. Additional insights into these phenomena were obtained by performing experiments in which the system was alternately operated with and without vibration at a feed flux of 17.2 L/m^2^/h, with results shown in Figure 5. The filtration was started without vibration, with the TMP increasing slowly from <10 kPa to more than 100 kPa when the filtrate flux was increased to 6.9 L/m^2^/h. At this point (*t* = 107 min), the vibration was turned on, with the flux kept constant at 6.9 L/m^2^/h, leading to a significant reduction in TMP from 114 to 6 kPa. The TMP then remained stable at 6 kPa over the last several data points (approximately 5 min). Thus, most of the increase in TMP observed in the absence of vibration was reversed by applying vibration.

The right‐hand axis in Figure 5 shows the effective resistance during the flux‐stepping experiment, evaluated as:

(2) $$R=\frac{TMP}{J}\;$$

where *J* is the filtrate flux. The resistance in the first step with vibration was *R* = 0.9 kPa/ L/m^2^/h, a value that is very similar to that obtained during the second stage under conditions where the filtration was performed with vibration throughout the experiment (data at 40% conversion in Figure 3). This suggests that the degree of fouling observed during the filtration in the absence of vibration could be fully reversed upon initiating the vibration, at least under the low‐fouling conditions explored in the first flux steps in Figure 5.

The filtrate flux was then increased step‐wise until obtaining 90% conversion in the presence of vibration, with the TMP remaining stable over each 25 min interval (although there was a small but distinct increase in the resistance in the flux‐step at 90% conversion). At this point, the vibration was turned off, with the TMP increasing rapidly from ≈30 kPa to more than 100 kPa over just 5 min of filtration. Re‐introducing the vibration instantly reduced the TMP, with the TMP decreasing from 105 to <35 kPa in just 15 min. The TMP then stabilized at a value of 32 kPa, which is within 10% of the TMP during the previous cycle at 90% conversion with vibration.

The vibration was then turned off again for 5 min, with the TMP rapidly increasing to more than 120 kPa in less than 5 min (with the conversion kept constant at 90%). Restarting the vibration was unable to restore the performance, with the TMP continuing to increase from 125 to 170 kPa in just 5 min. This suggests that once the fouling reaches a certain level the vibration ceases to be effective. Note that the membrane could still be cleaned with 0.5 N NaOH, with the resistance of the cleaned membrane (*R* = 1.3  ±  0.2 kPa/L/m^2^/h) almost equal to that of the fresh membrane before exposure to hIgG (*R* = 1.2 ± 0.1 kPa/L/m^2^/h).

### 3.2 Critical Flux/Conversion

**FIGURE 6:**
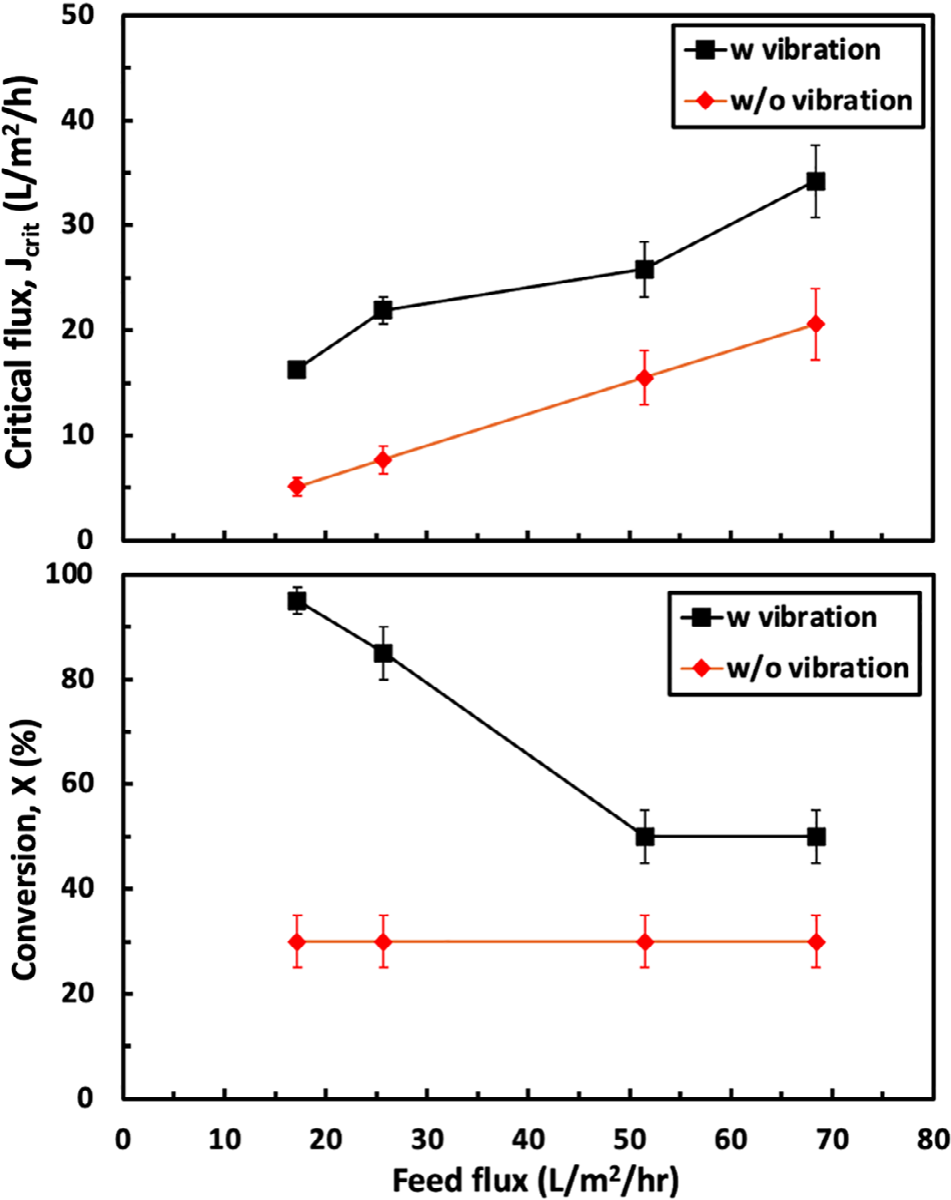
Critical flux (top) and maximum conversion (bottom) determined from flux‐stepping experiments performed using 10 g/L solutions of hIgG both with and without vibration.

Figure 6 shows the effects of the feed flux on both the critical flux and conversion determined from flux‐stepping experiments performed with a feed that initially contained a 10 g/L solution of hIgG, both with and without vibration. The data are plotted as the arithmetic mean of the flux values just before and after the TMP became unstable; the error bars represent the range between these flux values. The critical flux increases with increasing feed flux both with and without vibration, likely due to the reduction in concentration polarization. The data without vibration are nearly linear, leading to a constant value of the conversion of 30% ± 5%. In contrast, the conversion with vibration decreases as the feed flux increases, since the critical flux varies with feed flux in a less than linear fashion, that is, a 2‐fold increase in feed flux leads to a less than a 2× increase in critical flux. It was possible to achieve more than 90% conversion (more than a 10× concentration factor) when operating at a feed flux of 17.2 L/m^2^/h (1 mL/min feed flow rate) in the presence of vibration.

The large improvement in performance for the vibratory system is due in large part to the high shear rates generated near the membrane in the presence of vibration. The flow in the open channel cassette (in the absence of vibration) is classical parabolic flow, with the shear rate given as:

(3) $$\gamma =\frac{6q_{F}}{wh^{2}}$$

where *q_F_* is the feed flow rate, *w* is the width of the feed channel (*w* = 3.47 cm in the Vibro‐Lab 35P cassette), and *h* is the channel height (*h* = 0.17 cm). At a feed flow rate of 1 mL/min, Equation (3) gives a shear rate of γ  = 1 s^−1^. In contrast, the shear rate due to vibratory motion has been examined by Akoum et al. [17] and was given approximately as:

(4) $$\;\gamma_{vib}=2^{1/2}d{\left(\pi F\right)}^{3/2}\upsilon^{-1/2}$$

where *d* is the amplitude of vibration (*d* = 3.5 × 10^−3^ m), *F* is the vibration frequency (*F* = 26 Hz), and υ is the kinematic viscosity of the hIgG solution, which was evaluated as 1.4 × 10^−6^ m^2^/s for the 10 g/L solution using the Cannon‐Fenske viscometer. Equation (4) gives a shear rate of γ_*vib*_ ≈ 3000 s^−1^ independent of the feed flow rate. Thus, the observed increase in critical flux with increasing shear rate is not due to the increase in the effective shear rate (or mass transfer coefficient). Instead, this is likely due to the reduction in the hIgG concentration in the region near the retentate exit, with this lower hIgG concentration providing a greater driving force and, in turn, a large critical flux.

### 3.3 Performance of Open Channel Cassette vs. Screened Cassette

**FIGURE 7:**
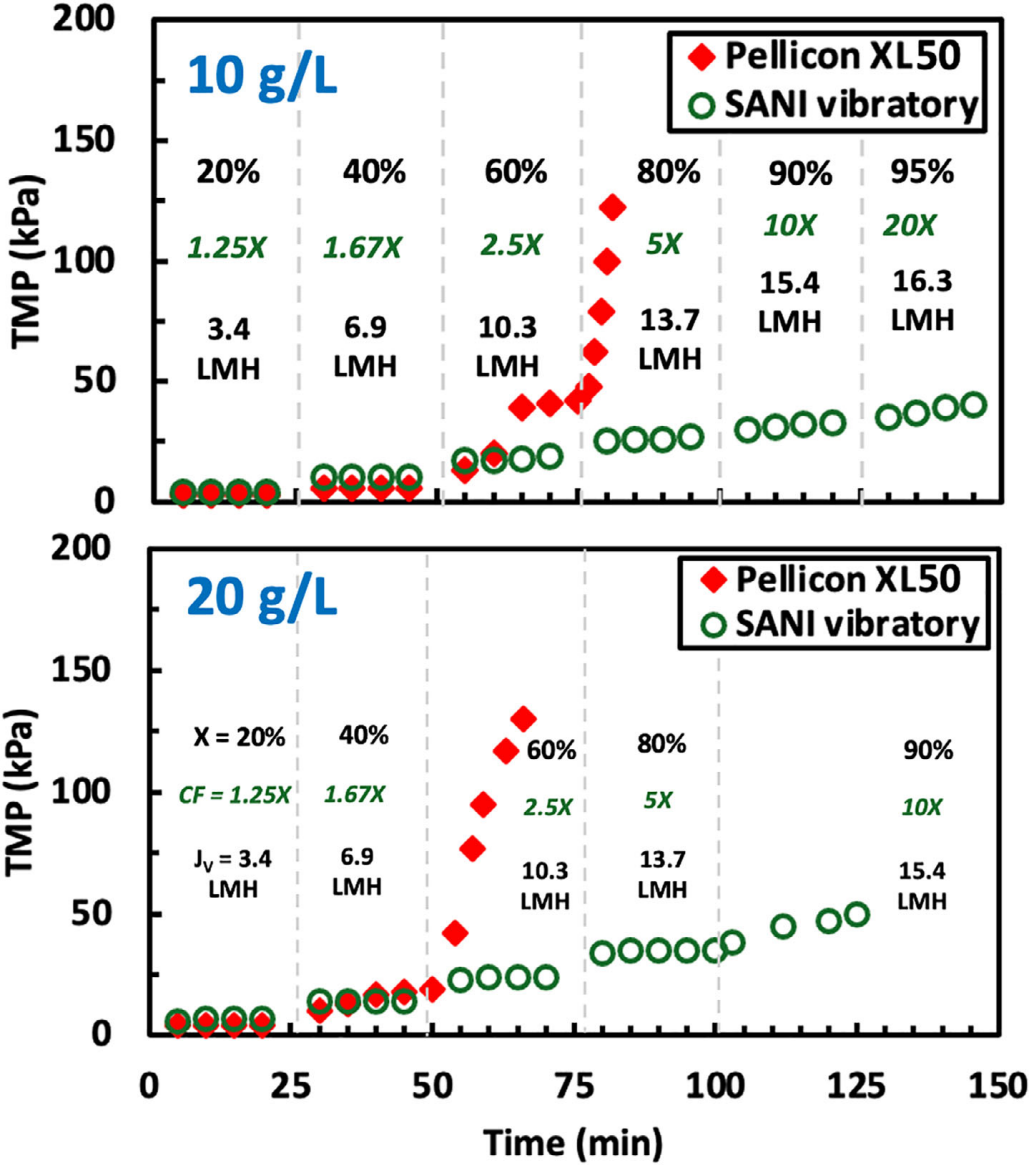
Flux‐stepping experiments performed with the SANI open‐channel cassette (with vibration) and the Pellicon XL50 cassette with A‐screen (without vibration), both with 30 kDa PES membranes at a feed flux of 17.2 L/m^2^/h. Top panel: initial hIgG feed concentration of 10 g/L. Bottom panel: initial hIgG feed concentration of 20 g/L.

Protein ultrafiltration, both as a batch operation and by SPTFF, is typically performed using screened‐channel cassettes since the screen (or spacer) enhances the mass transfer in the concentration polarization boundary layer adjacent to the membrane. Figure 7 shows data for flux‐stepping experiments performed using the Pellicon XL50 screened cassette and the open channel Vibro‐Lab 35P module, both with 30 kDa PES membranes operated at a feed flux of 17.2 L/m^2^/h with hIgG concentrations of 10 g/L (top panel) and 20 g/L (bottom panel). Only the Vibro‐Lab 35P was vibrated. For the 10 g/L hIgG feed, the Pellicon XL50 cassette shows an unstable TMP at 60% conversion with the maximum resistance increasing to 4.1 kPa/L/m^2^/h; this is more than twice the resistance of the Vibro‐Lab 35P at the same conversion (1.8 kPa/L/m^2^/h). The maximum concentration factor for the Pellicon XL50 cassette was only 2.5×, compared to the 20× concentration factor that could be achieved with the Vibro‐Lab 35P. Data with the 20 g/L hIgG feed showed similar results, with the maximum concentration factor increasing from 1.67× in the Pellicon XL50 to nearly 10× in the Vibro‐Lab 35P.

### 3.4 Long Term SPTFF Performance

Based on the data obtained in Figures 2 and 3, long‐term filtration experiments were performed to demonstrate the potential of using vibratory SPTFF for intensified/continuous processing without cleaning or replacement. Data were obtained using 800 mL of a 10 g/L solution of hIgG at a feed flux of 17.2 L/m^2^/h; the larger feed volume was used to minimize the number of pump passes since the process was performed in total recycle mode. Experiments were performed at 90% conversion, both with and without vibration (corresponding to a filtrate flux of 15.4 L/m^2^/h), and also at conversions of 88% and 95% in the presence of vibration. In each case, the initial resistance was *R* = 2.6 ±  0.1 kPa/L/m^2^/h, which is 2× larger than the resistance of the clean 30 kDa membrane (*R* = 1.3  ±  0.2 kPa/L/m^2^/h), due to fouling and/or concentration polarization associated with the hIgG.

**FIGURE 8:**
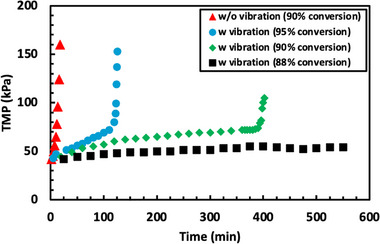
Long‐term filtration performed with 10 g/L hIgG solutions at a 17.2 L/m^2^/h feed flux for different conversions, both with and without vibration.

When the system was operated without vibration, the TMP became unstable almost immediately, increasing from 42 kPa to more than 150 kPa in just 20 min (Figure 8). This behavior is completely consistent with the low critical flux identified during the flux‐stepping experiments under these conditions (J_crit_ = 3.4 L/m^2^/h). The vibratory SPTFF experiment at 95% conversion showed a slow increase in TMP over the first 2 h, but this was followed by a rapid increase in TMP that required the system to be shut down before the pressure exceeded the maximum operating limit for the Vibro‐Lab 35P system. The period of stable operation could be increased to more than 6 h by reducing the conversion to 90%; the TMP remained stable throughout the full 9 h at a conversion of 88%. The hIgG concentration in the retentate exit stream measured at several time points during the run at 88% conversion was 82 ± 1 g/L, in excellent agreement with the expected concentration factor of 8.3×.

Interestingly, the TMP in the experiments performed with 90% and 95% conversion in the presence of vibration became unstable almost immediately after the TMP exceeded 75 kPa. This behavior is consistent with the results in Figure 3 where the run at a feed flow rate of 1 mL/min showed stable operation out to 95% conversion (with the TMP <75 kPa for the 6 flux steps) while the TMP at a feed flow rate of 3 mL/min became unstable at 60% conversion with the initial TMP at that flux step exceeding 75 kPa. The origin of this behavior is still to be determined, although it might be associated with the development of a very highly concentrated hIgG solution in the boundary layer above the membrane. This would also explain the continued decline in the feed concentration in Figure 3 during operation at 95% conversion, with the loss of hIgG arising from the accumulation of hIgG within the membrane module. Future studies will be needed to develop a more complete understanding of the onset of the critical flux in this system.

**FIGURE 9:**
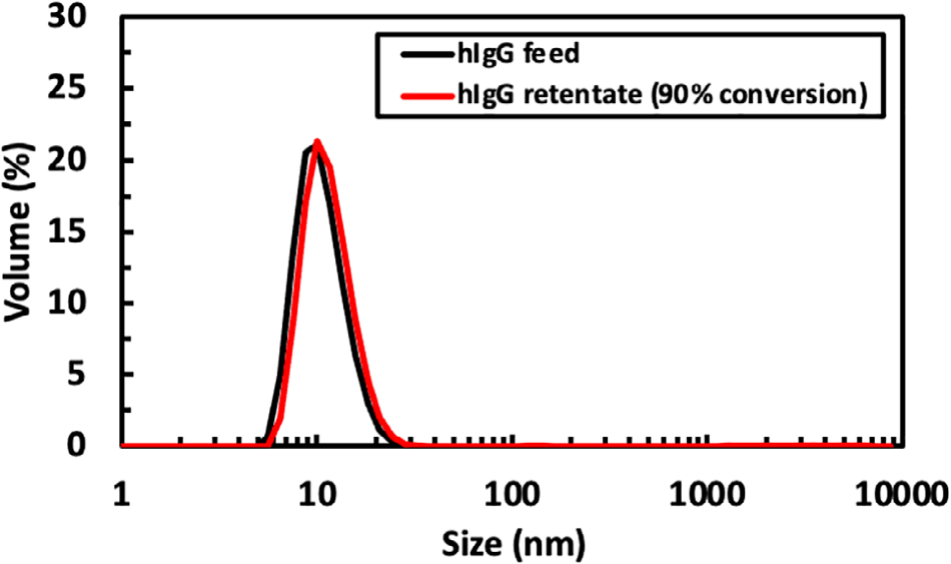
Particle size distributions determined by DLS for hIgG in the fresh feed (black curve) and in a retentate sample obtained during SPTFF at 90% conversion at *t* ≈ 350 min (red curve, sample obtained from experiment in Figure 8).

To verify that the vibratory SPTFF did not cause any aggregation or fragmentation of the hIgG, samples were taken from the line leaving the retentate exit during the SPTFF experiment at 90% conversion (at t ≈ 350 min) and analyzed using dynamic light scattering (DLS). Results are shown in Figure 9. The hIgG in the fresh feed exhibited a single peak with a *Z*‐average diameter of 10 nm; there were no detectable oligomers or higher‐order aggregates. The DLS profile for the retentate sample appears nearly identical, with no evidence of any hIgG aggregates or fragments formed during the SPTFF experiment. Future studies using monoclonal antibody products will include the use of size exclusion chromatography (SEC) to determine if there are any increases in low or high MW species (mAb fragments or dimers), including studies to assess mAb biological activity before and after SPTFF.

**FIGURE 10:**
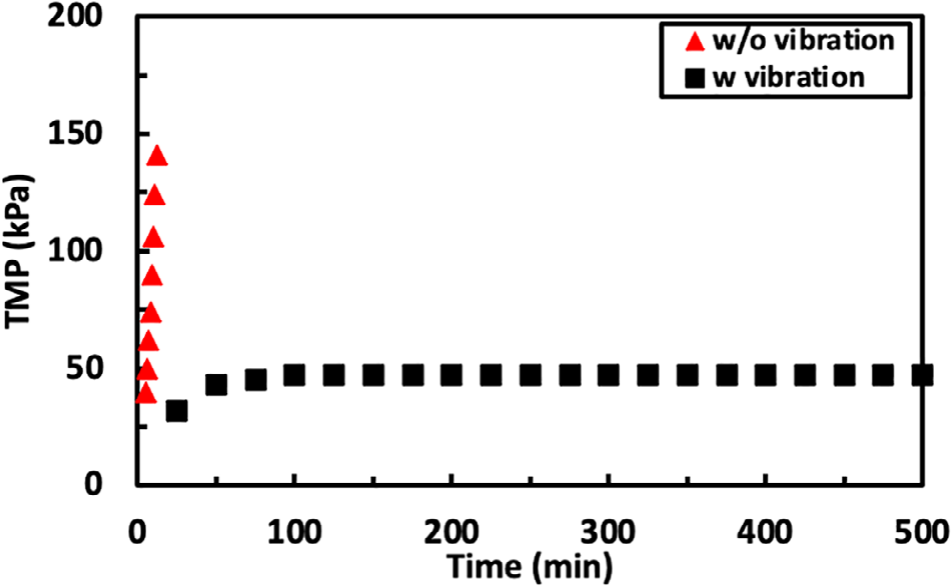
Long term filtration performed with the Vibro‐Lab 35P with 30 kDa PES membranes using 20 g/L hIgG feed solutions, both with and without vibration. Data were obtained at a feed flux of 17.2 L/m^2^/h.

Additional validation of the vibratory SPTFF process was obtained using a feed solution of 20 g/L hIgG. The system was operated at a feed flux of 17.2 L/m^2^/h with 80% conversion (filtrate flux of 13.7 L/m^2^/h) with results shown in Figure 10. The TMP for the SPTFF run without any vibration increased rapidly within 10 min, with the TMP exceeding 140 kPa. The SPTFF experiment performed with vibration showed a small increase in TMP over the first 75 min (from 32 to 45 kPa), but the TMP remained below 50 kPa throughout the >8 h experiment. The hIgG concentration in the retentate exit was 102 ± 3 g/L, in excellent agreement with the 5× concentration factor for this experiment.

Table 1 summarizes the performance of the single‐stage Vibro‐Lab 35P cassette with literature data for SPTFF of hIgG using both single and multistage cassettes. The results obtained in this study with a 10 g/L hIgG feed show much higher flux and concentration factor than that reported by Dizon‐Maspat et al. [4] using a system constructed with two Pellicon capsules. However, it should be noted that the steady‐state feed concentration in our study was somewhat below the initial feed concentration due to the accumulation of hIgG within the hold‐up volume in the module and tubing. Insufficient information is provided in the other studies examined in Table 1 to assess whether there were similar effects in those experiments. The critical flux in our study is similar to the operating flux obtained by Jabra et al. [14] using a multistage Cadence SPTFF module consisting of 7 cassettes in a 3‐2‐1‐1 configuration (representing the number of cassettes per stage), although Jabra et al. reported a maximum concentration factor of only 11×. The single‐stage Vibro‐Lab 35P had similar flux and concentration factor as the Pall Cadence module when processing a 20 g/L hIgG feed, but it provided significantly higher concentration factors than the 2‐cassette arrangement studied by Jabra et al. [14] and the 7‐cassette system examined by Dizon‐Maspat et al. [4]. These results clearly show the significant benefits that can potentially be achieved using vibratory‐assisted dynamic filtration for inline protein concentration by SPTFF.

**TABLE 1:** Comparison of SPTFF performance for inline concentration of hIgG.

Module	Membrane material	Feed concentration (g/L)^a^	Filtrate flux (L/m^2^/h)	Number of stages (cassettes)	Concentration factor	Reference
Vibro‐Lab 35P	PES‐30 kDa	10	16	1	8–20×	This work
Pall Cadence	RC‐30 kDa	10	16	7 (3‐2‐1‐1)	11×	[14]
Pellicon capsule	RC‐30 kDa	5‐15	< 1	2 (1‐1)	≈ 7.5×	[15]
Vibro‐Lab 35P	PES‐30 kDa	20	14	1	5×	This work
Pall Cadence	RC‐30 kDa	20	15	7 (3‐2‐1‐1)	4.5×	[14]
Pellicon 3	RC‐30 kDa	20	15	2 (1‐1)	1.8×	[14]
T‐series cassette	RC‐10 kDa	20	35	7 (3‐2‐1‐1)	< 2×	[4]

^a^ All experimental results shown in this table used hIgG except the studies from [4] which used a purified mAb.

## 4 Conclusions

The results obtained in this study demonstrate that vibratory‐assisted SPTFF can be effectively used for inline protein concentration, with data obtained using hIgG as a model for monoclonal antibody products. Vibratory SPTFF provided greater than a 5‐fold increase in the critical flux and an 8‐fold increase in concentration factor using the same feed and module compared to that evaluated in the absence of vibration. Additionally, vibration was able to effectively “clean” membranes that were fouled previously to a certain level under non‐vibratory conditions; this cleaning was not possible when the membranes became heavily fouled. The vibratory system also significantly outperformed the screened Pellicon XL50 cassette and showed equal or better performance than literature studies using more complicated staged module configurations.

Long‐term SPTFF experiments using the vibratory system at 90% conversion with 10 g/L hIgG and a feed flux of 17.2 L/m^2^/h showed stable performance for more than 6 h, without any evidence of protein aggregation or fragmentation as determined by DLS. However, it is important to note that the steady‐state feed concentration in our experimental system decreased significantly compared to the original feed concentration due to the large hold‐up volume in the module/tubing relative to that in the feed reservoir. In addition, the long term experiment with a 20 g/L hIgG feed provided stable filtration for more than 8 h, yielding an hIgG concentration in the retentate stream up to 100 g/L. The vibratory system is commercially available at multiple scales, including 35, 280, and 3500 cm^2^ membrane area for process development units and 2.5, 5.0, 10, 20, 40, and 80 m^2^ area for pilot‐scale and manufacturing [18], which would accommodate the wide range of process streams of interest. Future studies will be needed to demonstrate scalable performance of this technology using real monoclonal antibody feed streams.

## Author Contributions

**Km Prottoy Shariar Piash**: Conceptualization, Investigation, Data curation, Writing ‐ original draft. **Ziqiao Wang**: Investigation, Data curation. **Claire MacElroy**: Investigation, Data curation. **Andrew L. Zydney**: Conceptualization, Funding, Supervision, Writing ‐ review & editing.

## Conflicts of Interest

The author declares no conflicts of interest.

## Data Availability

Data will be made available on request.
